# Predictors and Long-Term Outcome of Ablation of Discrete Pre-potentials in Patients With Idiopathic Ventricular Arrhythmias Originating From the Aortic Sinuses of Valsalva

**DOI:** 10.3389/fcvm.2021.767514

**Published:** 2021-12-07

**Authors:** Hui-Qiang Wei, Xiao-Gang Guo, Gong-Bu Zhou, Qi Sun, Jian-Du Yang, Hai-Yang Xie, Jackson Liang, Shu Zhang, Shulin Wu, Jian Ma

**Affiliations:** ^1^Department of Cardiology, Guangdong Cardiovascular Institute, Guangdong Provincial People's Hospital, Guangdong Academy of Medical Sciences, Guangzhou, China; ^2^State Key Laboratory of Cardiovascular Disease, Arrhythmia Center, National Center for Cardiovascular Diseases, Fuwai Hospital, Chinese Academy of Medical Sciences and Peking Union Medical College, Beijing, China; ^3^Department of Cardiology, Peking University Third Hospital, Beijing, China; ^4^Division of Cardiovascular Medicine, Electrophysiology Service, University of Michigan Health System, Ann Arbor, MI, United States

**Keywords:** ventricular arrhythmia, catheter ablation, sinus of Valsalva, discrete pre-potential, outcome

## Abstract

**Background:** The predictability and long-term outcome of the discrete pre-potential (DPP) of idiopathic ventricular arrhythmias (VAs) arising from the aortic sinuses of Valsalva (ASV) have not been fully identified.

**Methods:** Of 687 consecutive patients undergoing ablation of outflow tract VAs, there were 105 (15.3%) patients with VAs originating from the ASV region who were included. Detailed mapping was performed within the ASV in all patients. Electrocardiographic, electrophysiological parameters, and long-term success rate were compared between patients with and without the DPPs.

**Results:** A DPP was recorded in 67 of 105 (63.8%) patients, including 38 left sinus of Valsalva (LSV)-VAs (38/105, 36.2%) and 29 right sinus of Valsalva (RSV)-VAs (29/105, 27.6%). The patients with DPPs had wider QRS duration (152 ± 17 vs. 145 ± 14 ms, *p* < 0.001). The average of earliest activation time was significantly earlier in patients with DPPs (−38.6 ± 8.5 vs. −27.7 ± 5.7 ms, *p* < 0.001). Mean time from the first lesion to elimination of VAs was shorter in patients with DPPs (2.3 ± 2.1 s vs. 4.9 ± 1.0 s, *p* < 0.001). A stepwise logistic multivariable analysis identified only younger age as a significant predictor of DPP (age ≤ 35.5 years predicted DPP with 92.9% positive predictive value). During a follow-up duration of 42.5 ± 22.3 months, 63 (94.0%) patients with DPPs and 30 (78.9%) patients without DPPs remained free of recurrent VAs (*p* = 0.027).

**Conclusion:** Discrete pre-potentials were observed in 63.8% of patients with VAs arising from the ASV. Ablation in patients with DPPs was associated with higher long-term success. DPPs were seen more commonly in younger (age ≤ 35.5 years) patients.

## Introduction

The right or left ventricular outflow tracts (RVOTs or LVOTs) are the most common sites of origin for idiopathic ventricular tachycardia (VT) or premature ventricular contractions (PVCs). Radiofrequency (RF) catheter ablation has been considered as a safe and effective method to treat outflow tract ventricular arrhythmias (VAs), and arrhythmias from these sites are being increasingly recognized (1–3). Several studies have reported that a discrete pre-potential (DPP) can sometimes be recorded at the successful ablation sites within the aortic sinuses of Valsalva (ASV), and the presence of the DPPs has been considered to be useful in guiding ablation within the ASV (4–6). However, there remains a paucity of comparative data on the prevalence and predictability of identifying DPPs during mapping and ablation of idiopathic VAs arising from the ASV. Consequently, the aim of this study was to identify the electrocardiographic and electrophysiological predictors of DPPs of idiopathic VAs arising from the ASV.

## Methods

The data that support the findings of this study are available from the corresponding author upon reasonable request.

### Study Population

**Figure 1:**
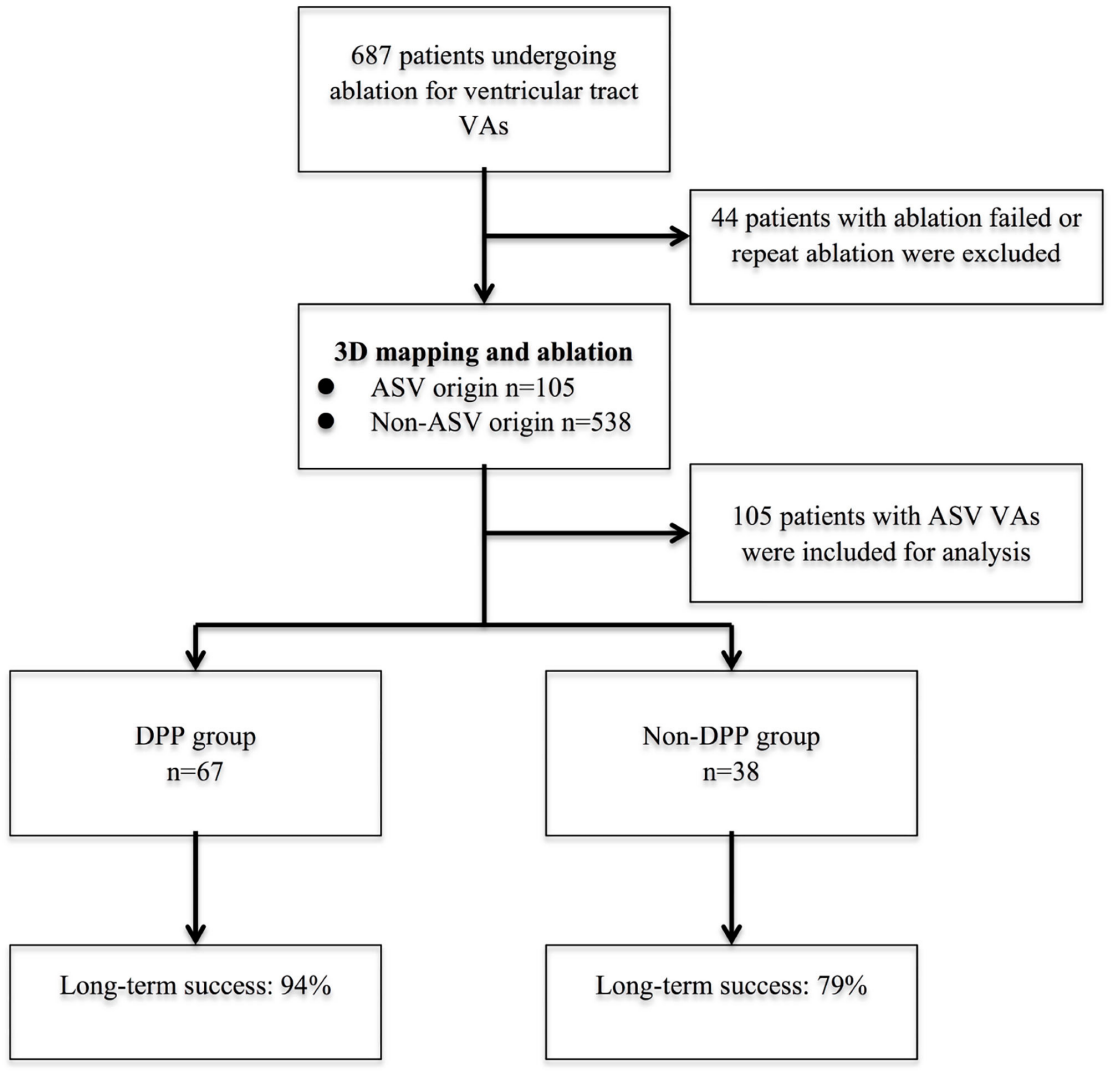
The flow diagram of the study and long-term success rate. 3D, 3-dimensional; ASV, aortic sinus of Valsalva; DPP, discrete pre-potential; VA, ventricular arrhythmia.

A total of 687 patients underwent ablation of ventricular outflow tract VAs between January 2012 and December 2018 at our center (Figure 1). Patients who underwent repeat ablation or ablation failed were excluded in this study (the anatomic location of their VA origin could not be determined). All patients had cardiac imaging with transthoracic echocardiography to confirm the absence of structural heart disease. All patients provided written informed consent before the catheter ablation. This study was approved by the institutional review board of Fuwai Hospital.

### ECG Analysis

Detailed analysis of clinical VT or PVCs was performed offline using an electrophysiological data acquisition system (LabSystem PRO, Bard Electrophysiology, Lowell, MA, USA). The following measurements were assessed during the clinical VT or PVCs using the measurement tool: (1) R- and S-wave amplitudes in leads I, II, III, and V_1_ to V_3_; (2) Q-wave amplitude in leads aVL, aVR, and the Q-wave ratio in lead aVL/aVR; (3) QRS duration; and (4) the precordial transitional zone (TZ); In addition, the precordial TZ was analyzed during the sinus rhythm. The TZ index was calculated as follows: TZ index = TZ score of the clinical VAs minus the TZ score of sinus rhythm (7). In cases with a QS pattern in lead I, we defined the R-amplitude as being 0 mV. The T-P segment was considered the isoelectric baseline for the measurement of R- and S-wave amplitude. All measurements were independently performed by two cardiac electrophysiologists.

### Electrophysiological Study

After the withdrawal of all antiarrhythmic drugs for at least five half-lives, all patients underwent electrophysiological evaluation. An electrophysiological study was performed with the patient in the fasting, non-sedated state. Catheters were inserted into the heart under fluoroscopy *via* the femoral vessels. If clinical VAs did not appear simultaneously, programmed stimulation or intravenous isoproterenol was performed to provoke the clinical arrhythmias. Bipolar signals were filtered at 30–500 Hz, and unipolar signals were filtered at 0.05–500 Hz. Intravenous heparin was administered to maintain an activated clotting time of 250 s.

### Mapping and Catheter Ablation

Three-dimensional electroanatomic mapping was applied in all patients using the CARTO mapping system (Biosense Webster, Inc., Diamond Bar, CA). Mapping and ablation were performed using a NaviStar ThermoCool catheter (Biosense Webster, Diamond Bar, California) *via* the femoral vessels. Point-by-point mapping was performed to create anatomic maps, and activation mapping was always used to identify the earliest activation site during the PVCs or VT as previously described (8). Pace mapping was also performed in some cases. An ideal target for ablation was selected based on the earliest activation time during the PVCs or VT. DPP was defined as sharp high-frequency potentials occurring after or during the local ventricular electrogram in sinus rhythm or preceding the local ventricular electrogram during PVCs that sometimes displayed double or multiple components.

When mapping and ablation were attempted in ASV, selective angiography through the irrigated catheter tip or aortic arteriography using a pigtail catheter was performed to locate the position of the ASVs. Detailed mapping in the ASV was achieved using the ablation catheter. RF energy was delivered using an initial power of 25 W with an irrigation rate of 17 mL/min. During RF delivery, if a decreased frequency or elimination of VAs occurred within the initial 15 s, the application was maintained and titrated for 60–90 s. If the earliest activation time of VAs was <20 ms, then additional mapping was performed below the valve, coronary venous system or retried in RVOT. After successful ablation, intravenous administration of isoproterenol and programmed ventricular stimulation were performed to reinduce clinical VAs. Acute procedural success was defined as elimination and non-inducibility of the clinical PVCs or VT with isoproterenol infusion and programmed stimulation after at least a 30-min waiting period after the last ablation lesion.

### Follow-Up

Continuous telemetric monitoring was performed for 24 h in all patients postablation. Long-term success was defined as no recurrence of the clinical VAs during a Holter monitoring. After hospital discharge, patients were seen in outpatient clinic every 3 months for the 1st year postablation, and every 6 months thereafter. All patients underwent 24-h Holter monitoring and transthoracic echocardiography during follow-up. If the patients had any rhythm-related symptoms, a 12-lead ECG or 24-h 12-lead Holter monitoring was performed to document the cause of the symptoms.

### Statistical Analysis

All continuous variables are expressed as mean ± SD. The categorical variables are presented as numbers and percentages. Categorical variables were compared using chi-squared analysis. Continuous variables were compared using the Student's *t*-test or Mann–Whitney *U*-test, depending on data distribution. Significant clinical characteristics and ECG parameters predicting predictors of DPPs were tested by univariately, and multivariable logistic regression models were constructed using a backward procedure with *p* > 0.1 as the default criterion for eliminating all variables. Independent variables were tested for collinearity, but no collinearity was present. Receiver operating characteristic (ROC) curves corresponding to the selected logistic regression models were constructed, and the area under the curve (AUC) was calculated to access the diagnostic value of the variables. The optimal cutoff equaled the maximum of Youden's index J, calculated from the ROC data as the cutoff with the greatest sum of sensitivity and specificity (J = sensitivity + specificity−1). A two-tailed value of *p* < 0.05 was considered statistically significant. All statistical analyses were performed using SPSS 19.0 (SPSS, Inc., Chicago, IL, USA).

## Results

### Baseline Characteristics

**Figure 2:**
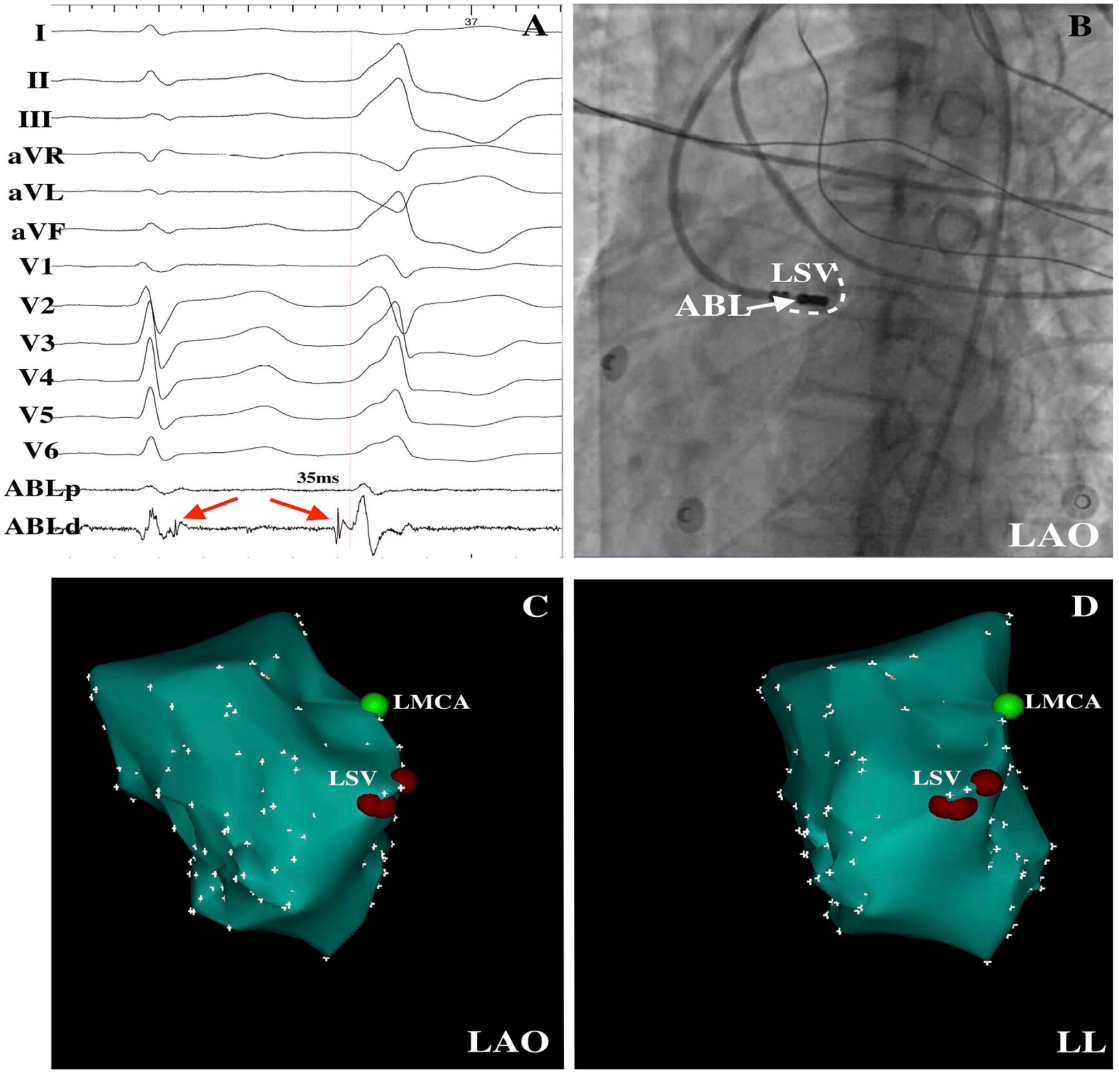
PVCs were successfully ablated within the LSV. **(A)** Delayed potential (red arrow) was observed during the sinus rhythm, and a DPP on the LSV preceded the QRS by 35 ms at the successful ablation site was recorded. **(B)** LAO radiographic view revealed the mapping catheter within the LSV. LAO **(C)** and LL **(D)** electroanatomical maps demonstrated the successful ablation points within the LSV. LSV, left sinus of Valsalva; DPP, discrete pre-potential; LAO, left anterior oblique; LL, left lateral; ABL, ablation catheter; LMCA, left main coronary artery.

**Figure 3:**
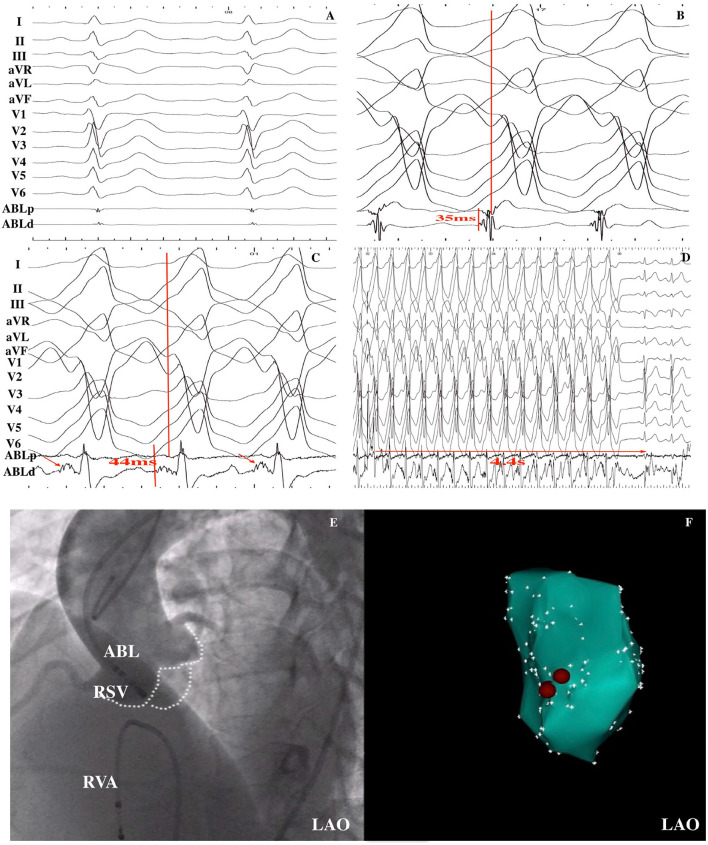
VT was successfully ablated within the RSV in a 15-year-old male patient. **(A)** Baseline 12-lead ECG was shown; **(B)** An early ventricular activation preceded by QRS by 35 ms during the VT was recorded at the RVOT, but ablation was ineffective. **(C)** Fragmented potential (red arrow) was found at the RSV, and the interval from the potential to QRS onset was 44 ms. **(D)** After RF energy delivery, VT was terminated after 4.4 s. **(E)** LAO fluoroscopic view of the mapping catheter at the successful ablation site as demonstrated by aortic artery angiography. **(F)** Electroanatomical CARTO map of the VT in the LAO view. Red points indicate ablation points. RSV, right sinus of Valsalva; RF, radiofrequency ablation; VT, ventricular tachycardia; LAO, left anterior oblique; ABL, ablation catheter; RVA, right ventricular apex.

**Figure 4:**
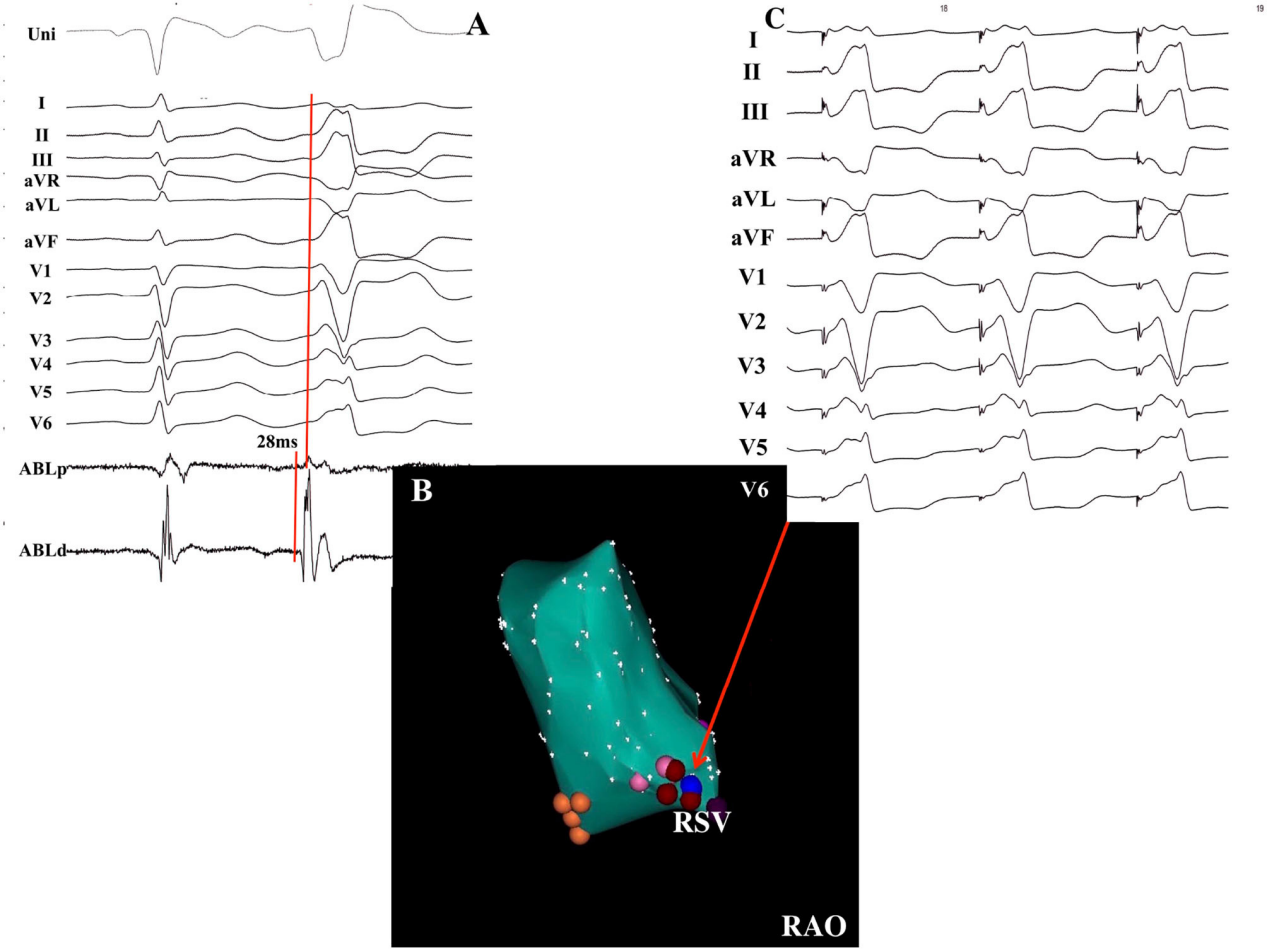
Successful ablation of PVCs without DPPs in a 63-year-old female patient. **(A)** The earliest activation recorded at the RSV preceded the QRS complex onset by 28 ms without DPPs. **(B)** Electroanatomical CARTO map of the PVCs in the RAO view. Red points indicate the ablation points. Blue point indicates the pacing site. Orange points indicate the non-coronary sinus of Valsalva. **(C)** Pacing mapping was excellent when pacing was delivered at the ablation site. RSV, right sinus of Valsalva; DPP, discrete pre-potential; RAO, right anterior oblique.

The successful ablation site was located within the ASV region in 105 (15.3%) of the 687 patients: 64 (61.0%) from the left sinus of Valsalva (LSV) and 41 (39.0%) from the right sinus of Valsalva (RSV). Of the 105 patients whose successful target site was from the ASV, a DPP was recorded in 67 of 105 (63.8%) patients, including 38 LSV-VAs (38/64, 59.4%) (Figure 2) and 29 RSV-VAs (29/41, 70.7%) (Figure 3). Baseline clinical and demographic data are presented in Table 1. Patients in whom DPPs were identified were younger in age than those without DPPs (37.6 ± 17.3 years vs. 52.2 ± 12.8 years, *p* < 0.001), and patients with DPPs were more likely to be male gender (62.7%) whereas those without DPPs were more likely to be female gender (57.9%) (*p* = 0.042) (Figure 4). In addition, patients with DPPs were more likely to have non-sustained or sustained VT rather than single PVC (20.9 vs. 2.6%, *p* = 0.01).

**Table 1:** Baseline clinical characteristics in patients with and without DPP.

	**DPP+ (*n* = 67)**	**DPP− (*n* = 38)**	***p*-value**
Age (years)	37.6 ± 17.3	52.2 ± 12.8	<0.001
Male	42 (62.7%)	16 (42.1%)	0.042
LVEF (%)	60.4 ± 7.5	61.3 ± 6.8	0.828
PVC burden on 24-h Holter monitoring	30,754 ± 14,740	28,466 ± 13,575	0.456
Clinical arrhythmias			0.01
Frequent PVCs	53 (79.1%)	37 (97.4%)	
Non-sustained or sustained VT	14 (20.9%)	1 (2.6%)	
Number of failed AADs	0.9 ± 0.8	1.2 ± 0.7	0.098
Structural heart disease	0	0	-

*Values are described as mean ± SD or n*.

*DPP, discrete pre-potential; LVEF, left ventricular ejection fraction; PVC, premature ventricular contraction; VT, ventricular tachycardia; AAD, antiarrhythmic drug*.

### ECG Characteristics

The VA ECG characteristics are summarized in Table 2. Patients with DPPs had wider QRS (152 ± 17 vs. 145 ±14 ms, *p* < 0.001). Q-wave amplitude in lead aVR was greater in patients with DPPs (0.94 ± 0.25 vs. 0.81 ± 0.16 mV, *p* < 0.001). S-wave amplitude in leads V_1_ and V_2_ was greater in patients with DPPs (1.03 ± 0.55 vs. 0.57 ± 0.40 mV, *p* < 0.001; 1.47 ± 0.91 vs. 0.90 ± 0.58 mV, *p* < 0.001, respectively). The TZ index was larger in patients with DPPs compared with those without DPPs (−0.53 vs. −1.22, *p* = 0.005).

**Table 2:** ECG parameters during the VAs between patients with and without DPP.

	**DPP+ (*n* = 67)**	**DPP− (*n* = 38)**	***p*-value**
QRS duration (ms)	152 ± 17	145 ± 14	0.019
R-wave amplitude in lead I (mV)	0.20 ± 0.25	0.15 ± 0.15	0.248
R-wave amplitude in lead II (mV)	1.82 ± 0.47	1.63 ± 0.37	0.105
R-wave amplitude in lead III (mV)	1.77 ± 0.61	1.63 ± 0.37	0.194
R-wave amplitude in lead aVF (mV)	1.79 ± 0.54	1.62 ± 0.31	0.117
R-wave ratio in lead II/III	1.10 ± 0.37	1.02 ± 0.17	0.260
Q-wave amplitude in lead aVL (mV)	0.86 ± 0.40	0.80 ± 0.29	0.361
Q-wave amplitude in lead aVR (mV)	0.94 ± 0.26	0.81 ± 0.16	<0.001
Q-wave ratio in lead aVL/aVR	0.96 ± 0.46	1.02 ± 0.39	0.496
R-wave amplitude in lead V_1_ (mV)	0.45 ± 0.32	0.39 ± 0.27	0.285
S-wave amplitude in lead V_1_ (mV)	1.03 ± 0.55	0.57 ± 0.40	<0.001
R-wave amplitude in lead V_2_ (mV)	0.91 ± 0.48	0.97 ± 0.51	0.478
S-wave amplitude in lead V_2_ (mV)	1.47 ± 0.91	0.90 ± 0.58	<0.001
R-wave amplitude in lead V_3_ (mV)	1.41 ± 0.65	1.65 ± 0.74	0.056
Precordial TZ index	−0.53	−1.22	0.005

*Values are displayed as mean ± SD*.

*DPP, discrete pre-potential; TZ, transitional zone*.

### Electrophysiological Characteristics

Detailed mapping of the ASVs was performed in all patients. The average of the earliest activation time was significantly earlier in patients with DPPs than in those without DPPs (−38.6 ± 8.5 vs. −27.7 ± 5.7 ms, *p* < 0.001). RVOT mapping was initially performed in 33 (49.3%) patients with DPPs and 12 (31.6%) patients without DPPs (*p* = 0.101). The mean time from the first RF lesion to the elimination of VAs was shorter in patients with DPPs than in those without DPPs (2.3 ± 2.1 vs. 4.9 ± 1.0 s, *p* < 0.001). The total number of RF applications in patients with DPPs was significantly less than in those without DPPs (2.1 ± 1.0 vs. 2.7 ± 0.9, *p* < 0.001) (Table 3).

**Table 3:** Electrophysiological characteristics during the VAs between patients with and without DPP.

	**DPP+ (*n* = 67)**	**DPP− (*n* = 38)**	***p*-value**
Mean activation time (ms)	−38.6 ± 8.5	−27.7 ± 5.7	<0.001
Mean time from the first RF lesion to the elimination of VAs (s)	2.3 ± 2.1	4.9 ± 1.0	<0.001
Total Number of RF application	2.1 ± 1.0	2.7 ± 0.9	<0.001
Initial mapping of right side	33 (49.3%)	12 (31.6%)	0.101

*Values are demonstrated as mean ± SD or n*.

*DPP, discrete pre-potential; RF, radiofrequency; VA, ventricular arrhythmia*.

### Predictors of DPP

**Figure 5:**
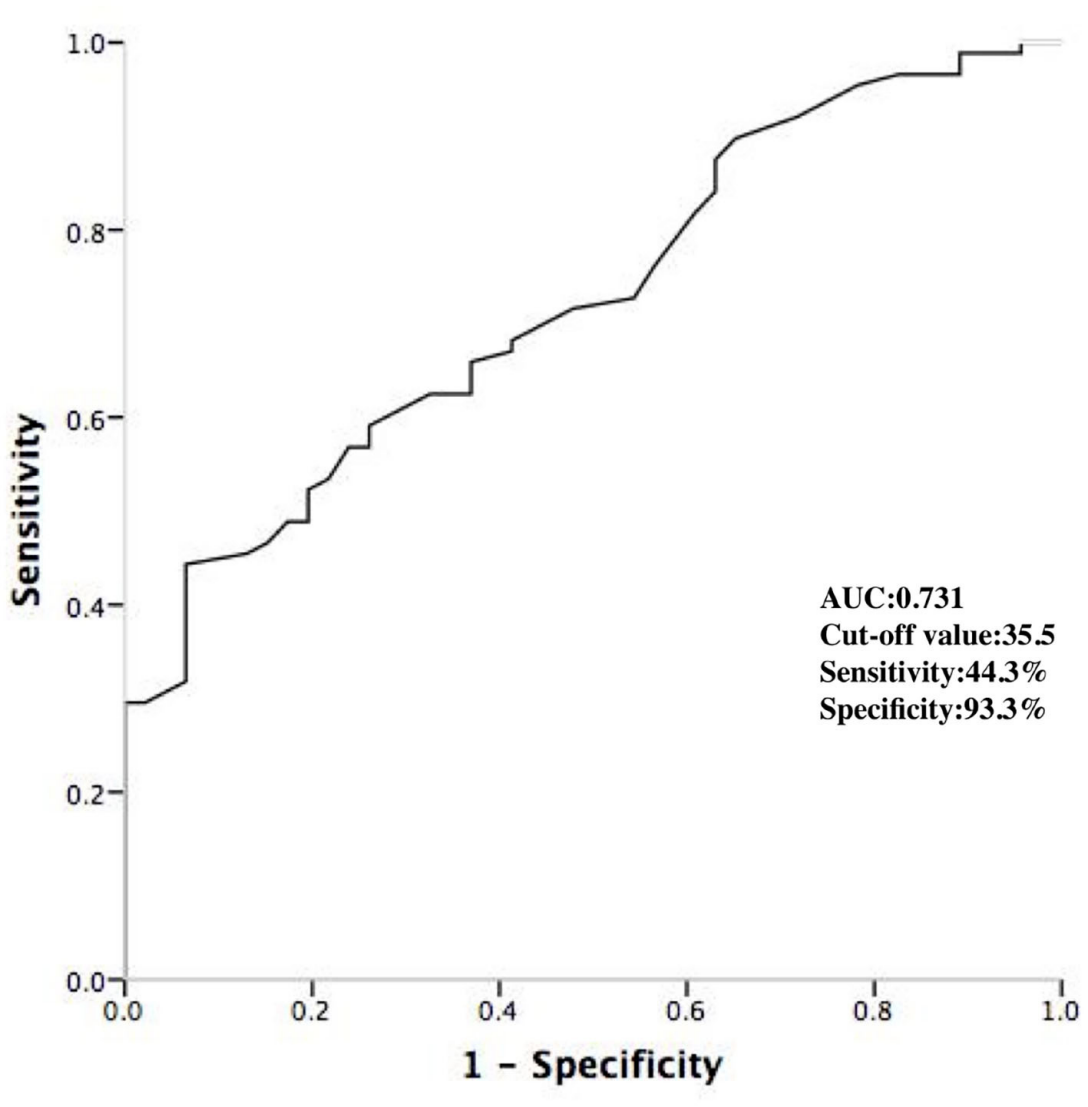
ROC curve analyzing the age to predict the DPPs. DPP, discrete pre-potential; AUC, area under curve.

A stepwise logistic multivariable analysis identified only the age as a significant predictor of a DPP (OR 1.61; 95% confidence interval 1.03–2.74; *p* < 0.001). On the basis of ROC curve analysis, an age of ≤ 35.5 years produced an AUC of 0.731 with a sensitivity of 44.3% and specificity of 93.3%, with a 92.9% of positive predictive value and a 46.7% of negative predictive value (Figure 5).

### Complications and Follow-Up

There were three peripheral vascular complications including two patients with pseudoaneurysm and one with arteriovenous fistula. There were no other major complications, including no coronary arterial damage or aortic valve injury in any patients. Over a total follow-up duration of 42.5 ± 22.3 months, 63 (94.0%) patients with DPPs and 30 (78.9%) patients without DPPs remained free of recurrent VAs in the absence of antiarrhythmic drugs. There was a significant difference in recurrent rate between patients with and without DPPs (6.0 vs. 21.1%, *p* = 0.027). Of these patients, Seven of 12 patients received repeat ablation. Successful ablation was achieved in the same location in two patients with DPPs. However, in the other five patients without DPPs, the distribution of ablation sites was as follows: RVOT in three patients, aorto-mitral continuity (AMC) in one patient, and below RSV-LSV commissure in one patient. Repeat ablation was successful in these five patients.

## Discussion

### Major Findings

To the best of our knowledge, this is the first study to investigate the predictors of DPPs of VAs originating from the ASV. In this study, we found that (1) a DPP can be recorded in 63.8% of the patients whose successful ablation site locates in the LSV or RSV; (2) RF ablation in patients with DPPs was associated with a higher long-term success rate compared with those without DPPs; (3) the age is the only independent predictor of DPPs; and (4) the age ≤ 35.5 years can predict the presence of DPPs with a 92.9% of positive predictive value.

### Presence of DPP

Previous studies have reported that extensions of the left ventricular myocardium frequently exist within the ASV (9–11). The fibrosis and fatty tissue among these muscular sleeves may create a possible VA substrate (11). The presence of DPPs of VAs originating from the ASV has been described in previous studies and considered to be useful in guiding ablation within the ASV (4–6). There has been a wide range in prevalence of DPPs during ASV and outflow tract VA ablation, ranging from 10% of all outflow tract VA ablations, 26% of all ASV ablations, and up to 100% of patients with ASV in a study by Ouyang et al. (4–6). In this study, DPPs were recorded in 67 (63.8%) of 105 patients in whom ablation from the ASV was successful in eliminating the VA. Non-sustained or sustained VT was commonly seen in the patients with DPPs, which is consistent with Ouyang's study. We surmise that the DPPs may be responsible for triggering the VA, similar to that observed from superior vena cava and pulmonary vein in patients with atrial fibrillation (12, 13). The low incidence of DPPs reported in previous studies may be due to decreased recognition of the low-amplitude DPP in some patients. The scale of the electrogram recorded in the ablation catheter should be magnified during the mapping within the ASV. In addition, small sample sizes may account for the low prevalence of the DPP.

### ECG Characteristics

Few studies have described the ECG characteristics of DPPs of VAs originating from the ASV. Hachiya et al. (4) reported nine of 35 patients with DPPs and no specific ECG findings were found. In this study, using quantitative ECG analysis, several unique ECG characteristics were found in patients with DPPs. The patients with DPPs were associated with wider QRS. Q-wave amplitude in lead aVR was greater in patients with DPPs. S-wave amplitude in leads V_1_ and V_2_ were greater in patients with DPPs. In addition, the TZ index was larger in patients with DPPs compared with those without DPPs. The extra-ventricular myocardium extensions may be responsible for these unique ECG characteristics. These myocardial extensions were asymmetrical, with sleeves routinely found along anterior portions of the RSV and LSV (14), which might produce a vector from anterior to posterior. This activation manner results in the terminal S-wave in leads V_1_ and V_2_.

### Predictors of DPP

The DPPs have been considered to be an indicator of successful ablation site within the ASV (4, 6). As Ouyang reported, two ventricular activation components were always recorded at the successful site in patients with VT originating from the ASV (6). In Ouyang's study, the mean age of the patients was 36.6 years. In this study, we found that the age was the independent predictor of DPPs. An age of ≤ 35.5 years produced an AUC of 0.731 with a sensitivity of 44.3% and specificity of 93.3%, with a high-positive predictive value, which was consistent with Ouyang's study. Younger patients were more likely to have a DPP, which might be explained by the fact that degenerative fibrosis of the myocardial extensions may occur over time with advancing age (15).

### Long-Term Clinical Outcome

Several investigators have demonstrated the favorable outcome in small series of patients with DPPs of VAs originating from the ASV (4–6). In this study, during a follow-up period of 42.5 ± 22.3 months, 94% of patients with DPPs remained free of recurrent VAs. However, a higher recurrent rate was observed in patients without DPPs. This may further prove that the DPPs represent the myocardial origin of VAs, and the presence of DPPs may help to identify successful ablation sites and improve long-term success. In some cases without DPPs, although successful ablation was initially achieved in ASC, the true arrhythmia origin may not be located in ASC due to the complexity of LVOT anatomy. The larger part of the RSV and the commissure between RSV and LSV are related to the interventricular septum which forms part of the subaortic valvular region and the RVOT septal wall. Similarly, the LSV is in close relationship with the LV summit, the interventricular vein, and the AMC (16). In our study, five patients without DPPs received repeat procedures. The final ablation sites were RVOT in three patients, AMC in one patient, and below RSV-LSV commissure in one patient. Therefore, careful mapping of all the neighbor structures around the ASC may be needed in some patients without DPPs undergoing the first procedure to reduce the recurrent rate.

### Study Limitations

This was a retrospective single-center study with a limited sample size. First, pace mapping was not routinely performed in all patients. Second, VAs from RSV-LSV junction were also defined as RSV or LSV-VAs and were not analyzed separately in this study. Furthermore, no non-sinus Valsalva cases were included because only four cases were found in our study, and the value of statistical analysis in such a small case series might be questioned. Third, although successful ablation was achieved within ASV in all cases, the real arrhythmia origin still could not be 100% sure. Final successful ablation in the ASV could not necessarily mean that the VAs originated from this spot. Furthermore, intracardiac echocardiography was not available in all patients due to the cost.

## Conclusion

Discrete pre-potentials were observed in 63.8% of the patients in whom VAs were successfully eliminated with ablation from the ASV region. Ablation in patients with DPPs was associated with higher long-term success. Younger age independently predicted the DPPs. A cutoff of 35.5 years of age indicated the presence of the DPPs with a 92.9% of positive predictive value.

## Data Availability Statement

The raw data supporting the conclusions of this article will be made available by the authors, without undue reservation.

## Ethics Statement

The studies involving human participants were reviewed and approved by Institutional Review Board of Fuwai Hospital. The patients/participants provided their written informed consent to participate in this study. Written informed consent was obtained from the individual(s) for the publication of any potentially identifiable images or data included in this article.

## Author Contributions

All authors listed have made a substantial, direct, and intellectual contribution to the work and approved it for publication.

## Funding

This study was supported by a grant from the National Natural Science Foundation of China (#81670309).

## Conflict of Interest

The authors declare that the research was conducted in the absence of any commercial or financial relationships that could be construed as a potential conflict of interest.

## Publisher's Note

All claims expressed in this article are solely those of the authors and do not necessarily represent those of their affiliated organizations, or those of the publisher, the editors and the reviewers. Any product that may be evaluated in this article, or claim that may be made by its manufacturer, is not guaranteed or endorsed by the publisher.
